# Treatment failure in a typhoid patient infected with nalidixic acid resistant S. enterica serovar Typhi with reduced susceptibility to Ciprofloxacin: a case report from Cameroon

**DOI:** 10.1186/1471-2334-5-49

**Published:** 2005-06-21

**Authors:** Njinkeng J Nkemngu, Etienne DN Asonganyi, Anna L Njunda

**Affiliations:** 1 St. John's Maternity and Hospital, Fiango, Kumba, Cameroon; 2 St. Joseph's medical Centre, Yoke-Muyuka, Cameroon; 3 University Teaching Hospital, University of Yaounde I, Yaounde, Cameroon; 4 Faculty of Health Sciences, University of Buea, Buea, Cameroon

## Abstract

**Background:**

Fluoroquinolones or third generation cephalosporins are the drugs of choice for the treatment of typhoid fever. Treatment failure with fluoroquinolones has been reported in Asia and Europe. We report a case of ciprofloxacin treatment failure in typhoid fever in Cameroon.

**Case presentation:**

A 29-year-old female patient with suspected typhoid fever from Kumba, Cameroon, yielded growth of Salmonella enterica serovar Typhi in blood culture. The isolate was resistant to nalidixic acid but sensitive to ciprofloxacin by disc diffusion test. However, the patient did not respond to treatment with ciprofloxacin, although the isolate was apparently susceptible to ciprofloxacin.

**Conclusion:**

Treatment failure with ciprofloxacin in our case indicates the presence of nalidixic acid resistant S. enterica serovar Typhi (NARST) with reduced susceptibility to ciprofloxacin in Cameroon (Central Africa).

## Case presentation

A 29-year-old woman from Kumba, Cameroon, was admitted in January 2004 to St. John's Hospital and Maternity, Kumba, with a five-day history of fever, emesis, poorly localized abdominal discomfort, myalgias and hepatosplenomegaly. Her total leukocyte count was 1.7 × 10^9^/l, (neutrophils 51%, lymphocytes 43%, monocytes 5%). Urinalysis was normal and thin and thick film examinations of the peripheral blood were negative for malaria. The patient also tested negative for HIV. A blood Widal test however, showed a titre of 80 against "O" (somatic) antigen and 160 against the "H" (flagella) antigen of Salmonella enterica serovar Typhi (recommended cut-off titre in our hospital: ≥ 1:80 and ≥ 1:160 for the "O" and "H" antigens respectively). Blood culture grew Salmonella enterica serovar Typhi. Two months prior to her illness, she had suffered from an attack of suspected typhoid fever and had been treated with chloramphenicol 500 mg every 6 hours for 14 days.

Antibiogram of the isolated S. enterica serovar Typhi was performed by disc diffusion techniques as recommended by NCCLS guidelines [1], Minimum inhibitory concentrations (MIC) of nalidixic acid and ciprofloxacin were determined by agar dilution method [2]. The antibiotic discs used included ampicillin 10 μg (Beecham), co-trimoxazole 1.25/23.75 μg (Roche), chloramphenicol 30 μg (Antibioticos SA), ciprofloxacin 5 μg (Bayer), nalidixic acid 30 μg (Sigma) and ceftriaxone 30 μg (Roche). The isolate was found resistant to nalidixic acid, ampicillin, co-trimoxazole and chloramphenicol, but susceptible to ceftriaxone and ciprofloxacin by disc diffusion test. The MICs of ciprofloxacin and nalidixic acid were 0.5 μg/ml and 32.0 μg/ml respectively. The patient remained febrile after 7 days of oral administration of 500 mg ciprofloxacin every 12 hours. Thereafter, the patient was administered 1 g ceftriaxone every 12 hours intravenously, which rendered her afebrile within four days. Treatment was continued for another 3 days. The patient did not relapse on follow-up.

## Conclusion

The emergence of multi-drug-resistant Salmonella enterica serovar Typhi (MDRST) strains was first reported in the 80 s, in Asia. Sporadic cases of ciprofloxacin treatment failure in typhoid fever have been reported in Europe and more recently, in Asia [3,4]. Our report indicates that MDRST and nalidixic acid resistant Salmonella enterica serovar Typhi (NARST) strains are now appearing in Cameroon, Central Africa. NARST have also been reported in East Africa [5]. However, treatment failure with fluoroquinolones in patients affected by the NARST strains in East Africa has not been described, although several reports suggest that the clinical response to fluoroquinolones in patients infected with NARST may be inferior to the response in those infected with nalidixic acid-susceptible strains [[4-11], this report].

There may be single or multi-mutations in the quinolone-resistance-determining region of either DNA gyrase (gyrA or gyrB or both) or DNA topoimerase IV (parC and parE or both) or both enzymes, which cause resistance of Salmonella enterica serovar Typhi strains to fluoroquinolone [5,9]. Resistance may also be due to other mechanisms such as decreased permeability and active efflux of the antimicrobial agents. Previous studies have shown that MDRST strains in East Africa were related to earlier drug-susceptible isolates but were unrelated to MDRST isolates from Asia. [10]. MDRST and NARST isolates in Central African may be unrelated to those earlier reported in Asia, Europe and east Africa [3-11].

Ceftriaxone is an alternative drug in cases of quinolone resistant typhoid fever. However, there have been reports of high-level resistance to ceftriaxone (MIC= 64 mg/l) in both Salmonella enterica serovar Typhi and Paratyphi A [3]. Third generation cephalosporins are also expensive (a treatment course with parental Ceftriaxone is six times more expensive compared to oral ciprofloxacin in Cameroon), and regularly not available. The efficacy of azithromycin, which was recently shown to be an effective alternative treatment for uncomplicated enteric fever due to MDRST, needs to be confirmed in patients with typhoid fever due to a NARST strain [12]. Improved hygienic conditions and effective surveillance methods to monitor newly emerged MDRST and NARST strains in Africa and other enteric fever endemic regions are of utmost importance.

## Authors' contributions

NJN conceived and coordinated of the study, drafted the manuscript, analyzed the microbial tests results. EDNA helped in the drafting of the manuscript and analysis of microbial test results. ALN helped to draft the manuscript. All authors read and approved the final manuscript.

## Competing interests

The author(s) declare that they have no competing interests.

## Financial support

None

## Pre-publication history

The pre-publication history for this paper can be accessed here:


